# Type I IFN Inhibits Alternative Macrophage Activation during *Mycobacterium tuberculosis* Infection and Leads to Enhanced Protection in the Absence of IFN-γ Signaling

**DOI:** 10.4049/jimmunol.1600584

**Published:** 2016-11-14

**Authors:** Lúcia Moreira-Teixeira, Jeremy Sousa, Finlay W. McNab, Egídio Torrado, Filipa Cardoso, Henrique Machado, Flávia Castro, Vânia Cardoso, Joana Gaifem, Xuemei Wu, Rui Appelberg, António Gil Castro, Anne O’Garra, Margarida Saraiva

**Affiliations:** * Microbiology and Infection Research Domain, Life and Health Sciences Research Institute, School of Health Sciences, University of Minho, and Life and Health Sciences Research Institute/3B’s PT Government Associate Laboratory, 4710 Braga/Guimarães, Portugal;; † Laboratory of Immunoregulation and Infection, The Francis Crick Institute, London NW1 1AT, United Kingdom;; ‡ Instituto de Investigação e Inovação em Saúde, Universidade do Porto, 4200 Porto, Portugal;; § Instituto de Ciências Biomédicas Abel Salazar, Universidade do Porto, 4050 Porto, Portugal;; ¶ National Heart and Lung Institute, Faculty of Medicine, Imperial College London, London SW3 6NP, United Kingdom; and; ‖ Instituto de Biologia Molecular e Celular, Universidade do Porto, 4150 Porto, Portugal

## Abstract

Tuberculosis causes ∼1.5 million deaths every year, thus remaining a leading cause of death from infectious diseases in the world. A growing body of evidence demonstrates that type I IFN plays a detrimental role in tuberculosis pathogenesis, likely by interfering with IFN-γ–dependent immunity. In this article, we reveal a novel mechanism by which type I IFN may confer protection against *Mycobacterium tuberculosis* infection in the absence of IFN-γ signaling. We show that production of type I IFN by *M. tuberculosis*–infected macrophages induced NO synthase 2 and inhibited arginase 1 gene expression. In vivo, absence of both type I and type II IFN receptors led to strikingly increased levels of arginase 1 gene expression and protein activity in infected lungs, characteristic of alternatively activated macrophages. This correlated with increased lung bacterial burden and pathology and decreased survival compared with mice deficient in either receptor. Increased expression of other genes associated with alternatively activated macrophages, as well as increased expression of Th2-associated cytokines and decreased TNF expression, were also observed. Thus, in the absence of IFN-γ signaling, type I IFN suppressed the switching of macrophages from a more protective classically activated phenotype to a more permissive alternatively activated phenotype. Together, our data support a model in which suppression of alternative macrophage activation by type I IFN during *M. tuberculosis* infection, in the absence of IFN-γ signaling, contributes to host protection.

## Introduction

Tuberculosis (TB), caused by *Mycobacterium tuberculosis* infection, is a leading cause of mortality and morbidity, causing ∼1.5 million deaths every year (1). Despite the efforts devoted to the understanding of this disease, mechanisms determining whether protection or pathogenesis results from *M. tuberculosis* infection remain poorly understood. An in-depth understanding of these mechanisms is critical for the design of novel preventive and therapeutic strategies based on the modulation of the immune response.

The initiation of the immune response to *M. tuberculosis* relies on bacterial recognition by pattern recognition receptors, such as TLR, by host sentinel cells (2). Recognition of *M. tuberculosis* triggers the production of key cytokines, chemokines, and antimicrobial molecules that are crucial to activate microbicidal mechanisms in innate immune cells and for the establishment of the adaptive immune response, restriction of bacterial growth, and, ultimately, host resistance (3–6). Among key cytokines produced by innate immune cells, IL-12 is critical for the induction of protective Th1 responses and IFN-γ production (5, 7–9). In turn, IFN-γ is crucial for full activation of macrophages, enhancing the production of cytokines and expression of microbicidal mediators, such as the inducible isoform of the enzyme NO synthase (NO synthase 2 [NOS2]) that is critical for controlling bacterial growth (9–13). Indeed, mice deficient in IFN-γ (*Ifng^−/−^* mice) or NOS2 (*Nos2^−/−^* mice) are extremely susceptible to *M. tuberculosis* infection, supporting the essential role of IFN-γ and NO in immunity against *M. tuberculosis* infection (10–14).

Different strains of *M. tuberculosis* interact with host TLRs in a distinct way, which is likely to shape the downstream immune response and disease outcome. We recently showed that, although most of the *M. tuberculosis* strains tested primarily activate TLR2, some activate TLR4 (15). TLR4 activation by *M. tuberculosis* was found to result in the expression of host-protective factors (e.g., TNF, IFN-γ, and NOS2) and to limit bacterial growth during in vivo infection (15). Despite the protective role of TLR4, a hypervirulent strain of *M. tuberculosis* recognized predominantly by this receptor was also found to induce high levels of type I IFN during infection (15), a cytokine that was associated with exacerbated disease (16, 17). Infection with other hypervirulent strains of *M. tuberculosis* showed a correlation between increased levels of type I IFN and increased virulence in mouse models of *M. tuberculosis* infection (18–20). *M. tuberculosis* infection of mice deficient in the type I IFN receptor (IFNAR) (*Ifnar^−/−^* mice) largely results in reduced bacterial load and/or increased survival compared with wild-type (WT) mice (19–22). Additionally, overexpression of type I IFN during *M. tuberculosis* infection provided robust evidence for the detrimental effects of type I IFN during TB (18, 23–26). Induction of high levels of type I IFN by direct instillation of type I IFN (18) or a type I IFN inducer (23, 25) into the lungs of *M. tuberculosis–*infected mice promoted disease severity. Abrogation of a negative regulator of type I IFN signaling increased host susceptibility to *M. tuberculosis* infection (24). Coinfection of mice with influenza A virus and *M. tuberculosis* resulted in increased bacterial loads in a type I IFN–dependent manner (26). Furthermore, a potential negative role for type I IFN was also revealed in human TB, because patients with active TB showed a prominent type I IFN–inducible blood signature (27–30) that correlated with the extent of radiographic disease (27) and that diminished upon successful treatment (30, 31). Thus, studies in mouse and humans highlight a potentially detrimental, rather than protective, role for type I IFN during TB (16–30).

The mechanisms that mediate type I IFN–dependent TB exacerbation are a major topic of investigation in the field (16, 17). Recent studies described that type I IFN suppresses the expression of protective proinflammatory cytokines (e.g., IL-1, TNF, and IL-12) while inducing the immune-suppressive cytokine IL-10 during *M. tuberculosis* infection (18–21, 32–34). Generation and trafficking to the lung of *M. tuberculosis*–permissive myeloid cells in response to induced type I IFN may also contribute to exacerbated disease (22, 23, 35). Type I IFN also was shown to impair Th1 cell responses in in vivo mouse models (18, 20, 32, 36) and to inhibit IFN-γ–induced antimicrobial responses in murine macrophages (33) and human monocytes (37, 38). The interplay between type I and type II IFN is further supported by a recent report by Desvignes et al. (39) describing an unanticipated protective role for type I IFN during *M. tuberculosis* infection in the absence of IFN-γ signaling. Mice deficient in both type I and type II IFNRs (*Ifngr^−/−^* × *Ifnar^−/−^* mice) showed increased pulmonary pathology and early mortality following *M. tuberculosis* infection compared with single type II IFNR–deficient (*Ifngr^−/−^*) mice (39). The investigators unmasked the involvement of type I IFN in the recruitment and/or survival of myeloid cells into the lungs and restriction of *M. tuberculosis* infection of these cells when IFN-γ signaling was absent (39). A putative protective role for type I IFN in the absence of IFN-γ signaling also was suggested in human TB based on the observation that administration of type I IFN, together with multidrug antimycobacterial treatment, had beneficial effects against disseminated *Mycobacterium avium* infection in a patient with IFN-γR deficiency (40).

In this article, we describe a novel mechanism for type I IFN in regulating macrophage activation during infection with a virulent strain of *M. tuberculosis* in the absence of IFN-γ signaling. Using a TLR4-activating virulent strain of *M. tuberculosis* that induces high levels of type I IFN, we detected increased levels of arginase 1 (*Arg1*) gene expression in the lungs, along with other genes associated with alternatively activated macrophages, when IFNAR signaling was abrogated in IFN-γR–deficient mice following in vivo infection. This correlated with increased lung bacterial loads and pathology, as well as increased Th2-associated cytokines and decreased TNF levels. Thus, our data indicate that suppression of alternative macrophage activation during *M. tuberculosis* infection by type I IFN confers protection against *M. tuberculosis* infection in the absence of IFN-γ signaling.

## Materials and Methods

### Ethics statement

All animal experiments were performed in strict accordance with the recommendations of the European Union Directive 2010/63/EU and were previously approved by the Portuguese National Authority for Animal Health (Direção Geral de Alimentação e Veterinária).

### Mice

C57BL/6 WT mice and knockout (KO) mice (all backcrossed ≥10 generations to the C57BL/6 background) were bred and housed at the Life and Health Sciences Research Institute (ICVS) (WT, *Tlr2*^−/−^ and *Tlr4*^−/−^ mice), Instituto de Investigação e Inovação em Saúde (WT, *Tnf*^−/−^, *Nos2*^−/−^ mice), and at The Francis Crick Institute, Mill Hill Laboratory (WT, *Ifnar*^−/−^ and *Ifngr^−/−^* mice). *Ifngr*^−/−^ and *Ifnar*^−/−^ mice were crossed to obtain double-KO (dKO) mutant mice (*Ifngr*^−/−^ × *Ifnar*^−/−^). Bones from *Il1r^−/−^* and *Ticam^−/−^* mice (and WT controls) were generously provided by Dr. Teresa Pais (Instituto de Medicina Molecular, Lisbon, Portugal) and by Dr. Luigina Romani (University of Perugia, Perugia, Italy), respectively. Mice were bred and maintained for experiments in accordance with the European Union Directive 2010/63/EU or the United Kingdom Home Office regulation and the Animal Scientific Procedures Act, 1986. For infections with *M. tuberculosis*, animals were housed under barrier conditions in the Animal Biosafety Level 3 facility at ICVS. Mice were matched for sex and age for use in experiments.

### Bacteria

*M. tuberculosis* strains H37Rv Pasteur and BTB 02-171 were kindly provided by Dr. Pere-Joan Cardona (Experimental Tuberculosis Unit, Barcelona, Spain) and Dr. Gunilla Källenius (Karolinska Institutet, Stockholm, Sweden), respectively. *M. tuberculosis* strains were grown in Middlebrook 7H9 liquid media for 7–10 d, diluted into Proskauer Beck medium with 0.05% Tween 80 for further expansion to mid-log phase, and frozen in 1-ml aliquots at −80°C, as previously described (15). All stocks were checked for endotoxin contamination using the *Limulus* assay (Sigma) and found to be negative.

### Bone marrow–derived macrophages

Bone marrow–derived macrophages (macrophages) were differentiated from bone marrow precursors cultured in complete DMEM (containing 10% FBS, 1% sodium pyruvate, 1% HEPES and 1% l-glutamine; all from Life Technologies) supplemented with 20% L929 cell-conditioned media, as previously described (15). Briefly, total bone marrow cells were cultured in microbiological petri dishes (Sterilin) and kept at 37°C and 5% CO_2_. Cells were fed on day 4 with 4 ml of complete DMEM containing 20% L929 cell-conditioned media. On day 7, macrophages were harvested, counted, and seeded into 24-well tissue culture plates (Nunc) at 0.5 × 10^6^ cells per well in culture medium. Cells were infected with *M. tuberculosis* strains at a multiplicity of infection (MOI) of 2. In some experiments, macrophages were stimulated with LPS (0.5 μg/ml; Sigma). When indicated, polymyxin B (5 μ/ml; Sigma), cycloheximide (10 μg/ml; Sigma), rIFN-β (2 ng/ml; PBL), or rIFN-γ (5 ng/ml; R&D Systems) was added at the time of infection. At specific time points postinfection, total RNA was isolated and/or supernatants were collected.

### Enumeration of intracellular *M. tuberculosis* in macrophages

To determine the number of intracellular *M. tuberculosis* CFU present in macrophages following in vitro infection, supernatants were removed, and macrophages were washed with PBS and lysed with saponin (Sigma). This suspension was serially diluted and plated onto Middlebrook 7H11 agar (BD Biosciences) plates containing OADC, and colonies were counted after 3 wk of incubation at 37°C. Differences in bacterial uptake among WT, *Tlr4^−/−^*, *Ifnar^−/−^*, and *Nos2*^−/−^ macrophages were monitored by enumerating CFU at 4 h postinfection, with no significant differences observed (data not shown).

### Experimental infection

Mice were infected with *M. tuberculosis* strains H37Rv or BTB 02-171 via the aerosol route using an inhalation exposure system (Glas-Col) calibrated to deliver ∼100–200 CFU to the lungs. The infection dose was confirmed by determining the number of viable bacteria in the lungs of three to five mice 3 d after the aerosol infection. For bacterial load determination, mice were euthanized by CO_2_ inhalation, and the lungs were aseptically excised and individually homogenized, followed by plating of serial dilutions of the organ homogenate on Middlebrook 7H11 agar (BD Biosciences) supplemented with OADC and Panta. CFU were counted after 3 wk of incubation at 37°C.

### Quantitative real-time PCR analysis

Total RNA from infected lungs or cultured macrophages was extracted with TRIzol Reagent (Invitrogen), according to the manufacturer’s instructions. cDNA was synthesized using the SuperScript First-Strand Synthesis System for RT-PCR (Thermo Scientific). Target gene mRNA expression was quantified by real-time PCR (Bio-Rad CFX96 Real-Time System with C1000 Thermal Cycler) and normalized to *Hprt1* or *Ubiquitin* mRNA levels. Target gene mRNA expression was quantified using SYBR Green (Thermo Scientific) and specific oligonucleotides (Invitrogen) for *Tnf* ([F] 5′-GCC ACC ACG TCT TCT GTC T-3′, [R] 5′-TGA GGG TCT GGG CCA TAG AAC-3′) and *Ubiquitin* ([F] 5′-TGG CTA TTA ATT ATT CGG TCT GCAT-3′, [R] 5′-GCA AGT GGC TAG AGT GCA GAG TAA-3′) or TaqMan primer probes (Applied Biosystems) for *Nos2* (Mm00440502_m1), *Arg1* (Mm00475988_m1), *Ym1* (Mm00657889_mH), *Fizz1* (Mm00445109_m1), *Il4* (Mm00445260_m1), *Il5* (Mm00439646_m1), *Il13* (Mm00434204_m1), *Il10* (Mm00439614_m1) and *Hprt1* (Mm00446968_m1).

### Histology

Whole lungs were perfused in situ with PBS. The right upper lobe of infected lungs was excised and fixed in 3.7% phosphate-buffered formalin for 1 wk. Then tissue was embedded in paraffin and cut into 3-μm-thick sections. Lung specimens were stained with H&E and subjected to microscopic morphometric analysis. Lung surface area of inflammation was measured using ImageJ software (version 1.50e; National Institutes of Health). Briefly, entire lung sections were analyzed, and the areas corresponding to the whole section and individual lesions were manually selected and measured. The percentage of total lung area involved with inflammation was calculated by dividing the cumulative area of inflammation by the total lung surface area for each sample.

### NOS2 detection by immunofluorescence

NOS2 was detected by immunofluorescence with a rabbit anti-mouse NOS2 primary Ab IgG (M-19; Santa Cruz Biotechnology) and an Alexa Fluor 488 goat anti-rabbit secondary Ab IgG (Invitrogen), as previously described (15). DAPI was used to detect nuclei. Images were acquired on an Olympus BX61 fluorescence microscope using Cell^P software.

### Measurement of NO production

NO production was quantified in cell culture supernatants by the Griess reaction. Absorbance was measured at 550 nm using a microplate reader (Bio-Rad), and NO concentration was assessed using a sodium nitrite standard curve (0–100 μM analyzed in duplicate).

### Arginase activity assay

ARG1 activity in lung homogenates was determined using the Arginase Activity Assay Kit (Sigma), according to the manufacturer’s instructions. Briefly, 1 × 10^6^ lung cells were lysed for 10 min in 10 mM Tris-HCl (Calbiochem) (pH 7.4) containing 1 μM pepstatin A, 1 μM leupeptin, and 0.4% (w/v) Triton X-100 (all from Sigma). Each sample was incubated for 2 h at 37°C in the presence of assay reagent and then incubated with urea reagent for 1 h at room temperature. Urea levels were subsequently detected and calculated according to the manufacturer’s instructions. ARG1 activity is expressed as U/l, and data are shown as fold induction relative to uninfected control mice.

### Cytokine determination by ELISA

TNF concentration in the supernatants of infected macrophages was determined by ELISA using a commercially available kit (eBioscience), according to the manufacturer’s instructions. Cytokine levels from uninfected cells were below the assay level of detection (20 pg/ml; data not shown).

### Flow cytometry

Myeloid cell populations from infected lungs were characterized using flow cytometry. For cell surface staining, Abs against CD11b (M1/70; eBioscience), CD11c (N418), Ly6G (1A8), and Ly6C (HK1.4; all from BioLegend) were used. For intracellular staining of NOS2, lung cells were fixed with 2% paraformaldehyde and permeabilized with 0.5% saponin (Sigma) before staining with anti-NOS2 Ab (CXNFT; eBioscience). Samples were acquired on a LSR II flow cytometer with FACSDiva software (BD Bioscience). Data were analyzed using FlowJo software (TreeStar).

### Statistics

Data are shown as the mean ± SEM. Statistical tests, as described in the figure legends, were used to compare experimental groups, with *p* < 0.05 considered significant. GraphPad Prism 6 (GraphPad) was used for data analysis and preparation of all graphs.

## Results

### Early and elevated *Nos2* expression induced by infection with a virulent TLR4-activating strain of *M. tuberculosis* results in protection and not pathology

**FIGURE 1.**
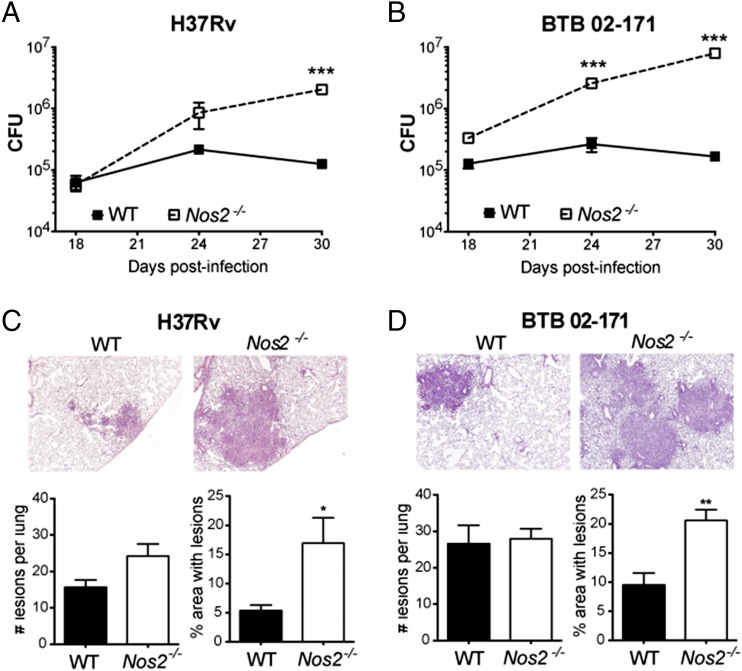
*Nos2* expression is essential for early control of virulent *M. tuberculosis* growth and immunopathology. WT and *Nos2*^−/−^ mice were infected with *M. tuberculosis* strains H37Rv (**A** and **C**) or BTB 02-171 (**B** and **D**). (A and B) At the indicated days postinfection, lung cell suspensions were prepared, diluted, and plated onto 7H11 agar to determine the number of mycobacterial CFU in the lungs. Data points show the mean ± SEM for five mice per group. ***p < 0.001, two-way ANOVA corrected for multiple comparisons with a Bonferroni test. (C and D) H&E-stained lung tissue from WT and *Nos2*^−/−^ infected mice was analyzed blindly at day 24 postinfection. Representatives of one of five animals are shown (original magnification ×4; upper panels). Morphometric analysis of the number and size of inflammatory lesions are also shown (lower panels). Each bar represents mean ± SEM for five mice per group. Data are representative of two independent experiments. *p < 0.05, **p < 0.01, unpaired *t* test.

Although it is generally recognized that NOS2 plays an essential role in the host defense against some strains of *M. tuberculosis* (12, 14, 41, 42), other studies assign a detrimental role to NOS2 in the context of *M. avium* infection (43) and other bacterial infections (44, 45). Previous data from our group showed that TLR4 activation by *M. tuberculosis* BTB 02-171 induced an early and elevated expression of *Nos2* in the lungs of infected mice that was accompanied by enhanced lung pathology; this was not seen when the TLR2-activating laboratory reference strain H37Rv was used (15). To investigate whether early and elevated levels of *Nos2* expression could explain the enhanced pathology, WT and *Nos2*^−/−^ mice were aerosol infected with *M. tuberculosis* strain H37Rv or BTB 02-171. As expected, bacterial loads were significantly increased in the lungs of H37Rv-infected *Nos2^−/−^* mice by day 30 postinfection, but no major differences in lung bacterial loads were observed at earlier times post-H37Rv infection (days 18 and 24 postinfection) compared with WT mice (Fig. 1A). However, higher bacterial loads were detected in the lungs at these early times, in addition to day 30, postinfection with BTB 02-171 in *Nos2^−/−^* mice compared with WT mice (Fig. 1B), in keeping with an earlier and elevated induction of *Nos2* transcription and NOS2 expression (15) (Supplemental Fig. 1). Increased bacterial loads observed in the absence of NOS2 were accompanied by enhanced lung pathology, which was quantified by the extent of lung area with inflammatory lesions at day 24 postinfection (Fig. 1C, 1D), confirming the protective, rather than detrimental, role of early and elevated *Nos2* levels observed during BTB 02-171 *M. tuberculosis* infection.

### TLR4 recognition of a virulent *M. tuberculosis* strain induces high *Nos2* transcription and NO production by infected macrophages

**FIGURE 2.**
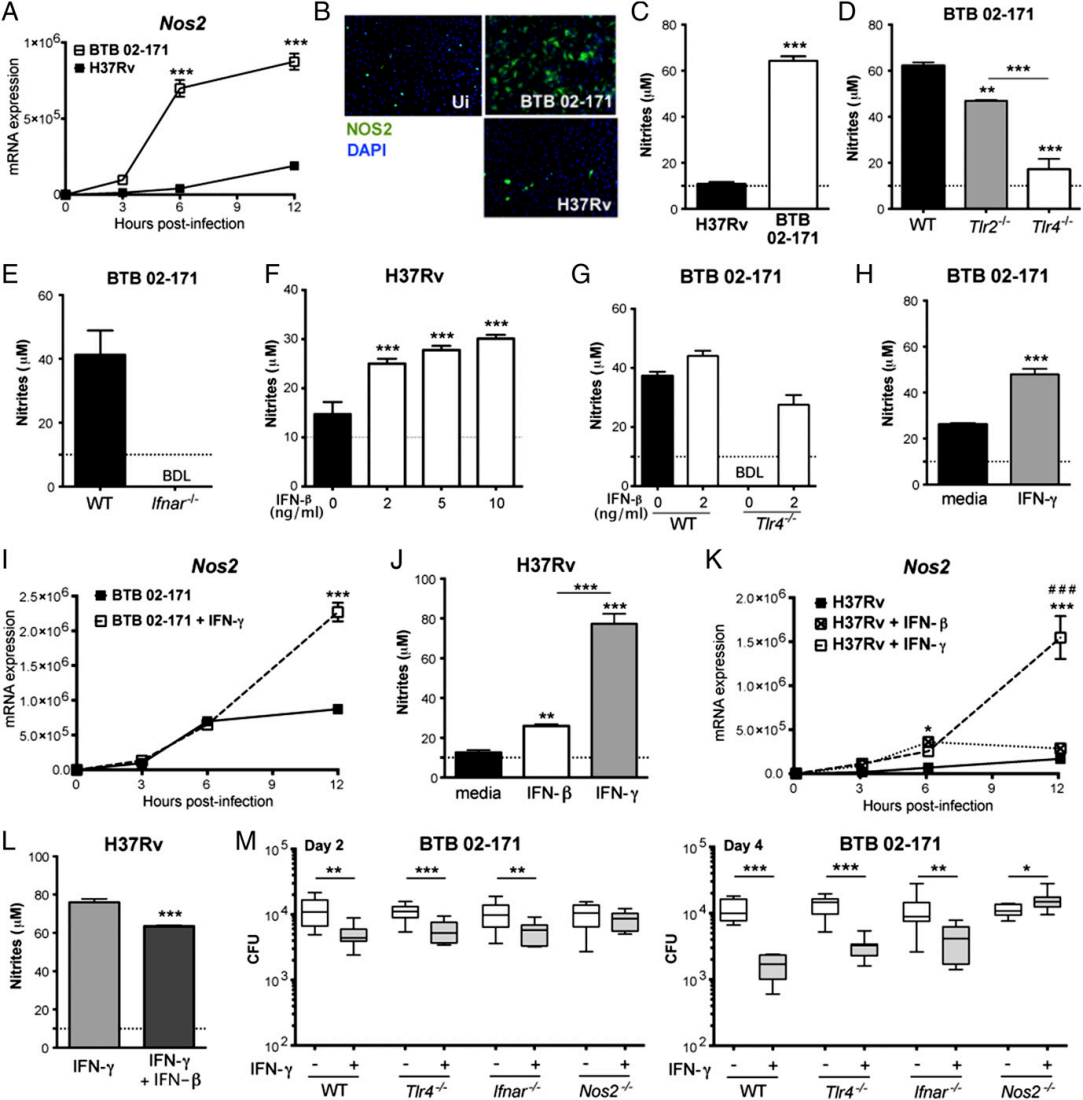
Type I IFN induces *Nos2* expression and NO production by *M. tuberculosis*–infected macrophages. (**A**–**C**) WT macrophages were infected with *M. tuberculosis* strains H37Rv or BTB 02-171 (MOI = 2). (A) At the indicated hours postinfection, *Nos2* mRNA levels were determined by quantitative real-time PCR and normalized to the expression of *Hprt1*. (B) Expression of NOS2 by uninfected (Ui) or infected macrophages was determined by immunofluorescence at 8 h (green signal indicates NOS2 stain, and blue signal indicates cell nuclei). (C) NO production was determined by Griess reagent assay of nitrites in culture supernatants at 24 h. WT, *Tlr2^−/^*,*^−^* and *Tlr4^−/−^* (**D**) or WT and *Ifnar^−/−^* (**E**) macrophages were infected with BTB 02-171, and NO levels in culture supernatants were determined by Griess reagent assay at 24 h. (**F**) WT macrophages were infected with H37Rv in the presence of increasing concentrations of rIFN-β, and NO levels in culture supernatants were determined by Griess reagent assay at 24 h. (**G**) WT and *Tlr4^−/−^* macrophages were infected with BTB 02-171 in the presence or absence of rIFN-β, and NO levels in culture supernatants were determined by Griess reagent assay at 24 h. WT macrophages were infected with BTB 02-171 (**H** and **I**) or H37Rv (**J** and **K**) in the presence or absence of rIFN-β or rIFN-γ. (H and J) NO levels in culture supernatants were determined by Griess reagent assay at 24 h. (I and K) *Nos2* mRNA levels were determined at the indicated hours postinfection. (**L**) WT macrophages were infected with H37Rv in the presence of rIFN-γ alone or rIFN-γ plus rIFN-β, and NO levels in culture supernatants were determined by Griess reagent assay at 24 h. Graphs show mean ± SEM of triplicate samples. (**M**) WT, *Tlr4^−/−^*, *Ifnar*^−/−^, and *Nos2^−/−^* macrophages were infected with BTB 02-171 (MOI = 0.5) in the presence or absence of rIFN-γ. Media were removed at 4 h postinfection, cells were washed in PBS, and fresh media was replaced. At day 2 (left panel) and day 4 (right panel) postinfection, cells were washed in PBS and lysed in 0.2% saponin, and bacterial loads were enumerated. Graphs show minimum to maximum CFU per well (five wells per experiment). Data are representative of at least two independent experiments. Significance was determined using two-way ANOVA corrected for multiple comparisons with a Bonferroni test (A, I, and K), an unpaired *t* test (C, H, L, and M), or one-way ANOVA with Bonferroni correction test (D, F, and J). Significance is relative to control group except in (K), where significance is shown relative to H37Rv (*) or to H37Rv + IFN-β (^#^). *p < 0.05, **p < 0.01, ***p < 0.001, ^###^*p* < 0.001. BDL, below detection level.

To investigate the mechanisms underlying high *Nos2* induction, we infected mouse bone marrow–derived macrophages (macrophages) with *M. tuberculosis* strain H37Rv or BTB 02-171. *Nos2* mRNA, NOS2 protein, and NO secretion were strongly upregulated upon macrophage infection with *M. tuberculosis* strain BTB 02-171, but not H37Rv (Fig. 2A–C), despite similar infection levels (Supplemental Fig. 2A), thus replicating the in vivo results. These data suggest that the higher induction of *Nos2* by BTB 02-171 may be a direct response of the macrophage to bacterial ligands, which is consistent with the fact that these phagocytes are among the first cells to recognize *M. tuberculosis* (4) and are well known for their capacity to upregulate *Nos2* and produce NO as a bactericidal mechanism (46). Because TLRs are capable of differential recognition of *M. tuberculosis* strains (15), we infected WT and *Tlr2^−/−^* and *Tlr4^−/−^* macrophages with the BTB 02-171 strain and measured NO secretion by quantifying the amount of nitrites in the supernatant 24 h later. TLR2 deficiency led to a minor reduction in nitrite concentrations; however, the lack of TLR4 nearly completely abrogated NO production (Fig. 2D). In line with this, *Nos2* transcription was also greatly reduced at 6 h postinfection in *Tlr4^−/−^* and *Ticam^−/−^* macrophages, but not in *Tlr2^−/−^* macrophages, compared with WT controls (Supplemental Fig. 2B, 2C). The upregulation of *Nos2* expression and NO production was a direct effect of the mycobacteria, because production of NO by BTB 02-171–infected macrophages was not affected by a concentration of polymyxin B (an endotoxin inhibitor) that completely abrogated NO production by macrophages stimulated with 500 ng/ml of LPS (Supplemental Fig. 2D). TLR4-dependent *Nos2* induction and NO production by infected macrophages were also observed postinfection with *M. tuberculosis* strain Harlingen (Supplemental Fig. 2E, 2F), which also was described as a TLR4-activating strain (15). These data suggest that TLR4 activation and the downstream TRIF pathway are required to initiate the signaling cascade involved in the upregulation of *Nos2* and NO production by macrophages infected with certain strains of *M. tuberculosis* that signal through TLR4 and are known to induce type I IFN production by infected macrophages.

### Type I IFN induces NO production by *M. tuberculosis*–infected macrophages

Our data showed that some *M. tuberculosis* strains induce high levels of *Nos2* transcription and NO production by macrophages by activating TLR4, whereas other strains only activate macrophages via TLR2. To investigate whether de novo protein synthesis was required for maximal *Nos2* induction by the TLR4-activating strains, WT macrophages were infected with *M. tuberculosis* BTB 02-171 strain in the absence or presence of cycloheximide (an inhibitor of protein biosynthesis), and *Nos2* transcription was measured 6 h postinfection. Blockade of de novo protein synthesis strongly inhibited *Nos2* induction following macrophage infection with the BTB 02-171 strain (Supplemental Fig. 2G), indicating that specific mediators produced upon TLR4 stimulation were required to induce *Nos2* transcription in infected macrophages. Thus, we investigated whether macrophage-derived type I IFN might induce the production of NO in response to *M. tuberculosis* by infecting WT and *Ifnar^−/−^* macrophages with the TLR4-activating BTB 02-171 strain. We found that NO production was completely abrogated and *Nos2* transcription was greatly inhibited in the absence of type I IFN signaling (Fig. 2E, Supplemental Fig. 2H). Other candidate mediators produced downstream of TLR4 activation and with a described role in the induction of NOS2 were also tested. Absence of IL-1R signaling did not affect *Nos2* transcription and only slightly reduced NO production by macrophages upon BTB 02-171 infection (Supplemental Fig. 2I, 2J). Partial decreases in the expression of *Nos2* and NO production were detected in the absence of TNF (Supplemental Fig. 2K, 2L), suggesting that TNF, although able to modulate levels of these mediators, was not absolutely required to induce NO production by BTB 02-171–infected macrophages. Absence of IL-10 slightly reduced *Nos2* transcription, but it did not affect NO production by macrophages upon BTB 02-171 infection (Supplemental Fig. 2M, 2N).

In further support of a role for type I IFN, addition of increasing concentrations of rIFN-β to macrophages infected with the TLR2-activating H37Rv laboratory strain resulted in significantly increased NO production (Fig. 2F). Although NO production by BTB 02-171–infected WT macrophages was not further enhanced by IFN-β, NO production by *Tlr4^−/−^* macrophages was rescued by the addition of IFN-β (Fig. 2G). Altogether, our findings indicate that TLR4-dependent induction of type I IFN resulted in the induction of NO production by macrophages in response to BTB 02-171 infection.

### Type I and II IFNs differentially regulate Nos2 induction in *M. tuberculosis*–infected macrophages

IFN-γ is known as the major *Nos2* regulator in *M. tuberculosis* infection (11, 13, 47). We have now shown that the induction of *Nos2* transcription in *M. tuberculosis*–infected macrophages may occur independently of IFN-γ, via type I IFN signaling (Fig. 2, Supplemental Fig. 2). Because both IFNs can induce *Nos2* transcription, we next investigated their relative contribution to NO production in response to *M. tuberculosis* infection. We infected WT macrophages with the BTB 02-171 strain in the presence of rIFN-γ at the time of infection. Enhanced NO production was observed in infected macrophages treated with IFN-γ (Fig. 2H). *Nos2* mRNA levels were also higher in infected macrophages treated with IFN-γ compared with untreated macrophages at 12 h (Fig. 2I), although similar levels were detected at earlier times postinfection (3 and 6 h). These findings suggest that both IFNs, macrophage-derived type I IFN and exogenous IFN-γ, may cooperate for maximal *Nos2* induction or that IFN-γ may induce higher levels of *Nos2* than type I IFN upon *M. tuberculosis* infection. To distinguish between these two possibilities, we infected WT macrophages with the H37Rv strain and added IFN-β or IFN-γ at the time of infection. Although IFN-β and IFN-γ induced NO production by H37Rv-infected macrophages, IFN-γ was significantly more potent than maximal doses of IFN-β (Fig. 2J). Similar levels of *Nos2* mRNA were induced by IFN-β and IFN-γ early postinfection (up to 6 h); however, although we observed a plateau in *Nos2* levels between 6 and 12 h postinfection in the presence of IFN-β, IFN-γ continued to increase the levels of *Nos2* transcription after 6 h postinfection (Fig. 2K). We showed previously that type I IFN impairs IFN-γ’s effects on cytokine production by *M. tuberculosis*–infected macrophages (33). Therefore, we examined whether type I IFN might affect IFN-γ’s activation of NO production by infected macrophages. WT macrophages were infected with the H37Rv strain and concomitantly treated with IFN-γ alone or IFN-γ plus IFN-β. NO production was reduced slightly when both IFN-γ and IFN-β were added to macrophages compared with IFN-γ alone (Fig. 2L), suggesting that, although type I IFN itself can stimulate NO production by *M. tuberculosis–*infected macrophages, it can also impair IFN-γ–dependent induction of NO production.

We next investigated whether type I IFN–induced *Nos2* could mediate restriction of *M. tuberculosis* in infected macrophages. WT, *Tlr4^−/−^*, *Ifnar*^−/−^, and *Nos2^−/−^* macrophages were infected with the BTB 02-171 strain, in the presence or absence of IFN-γ, and bacterial loads were assessed 2 and 4 d postinfection (Fig. 2M). Similar bacterial loads were detected at days 2 and 4 postinfection among WT, *Tlr4^−/−^*, *Ifnar*^−/−^, and *Nos2^−/−^* macrophages in the absence of IFN-γ (Fig. 2M). This suggests that TLR4 or type I IFN signaling each did not induce bacterial clearance by infected macrophages in vitro. IFN-γ treatment induced a significant reduction in bacterial load in WT, *Tlr4^−/−^*, and *Ifnar*^−/−^ macrophages but not in *Nos2^−/−^* macrophages (Fig. 2M). Thus, *Nos2*-dependent bacterial clearance by infected macrophages required the presence of IFN-γ. Altogether, our data highlight a differential regulation of *Nos2* transcription in *M. tuberculosis*–infected macrophages by type I and type II IFNs; IFN-γ is a more potent inducer of NO production, by sustaining increased *Nos2* transcription for longer times postinfection, and this seems to be crucial for efficient bacterial clearance by macrophages.

### Type I IFN controls bacterial growth and immunopathology in the lungs of mice infected with a virulent *M. tuberculosis* strain in the absence of IFN-γ signaling

**FIGURE 3.**
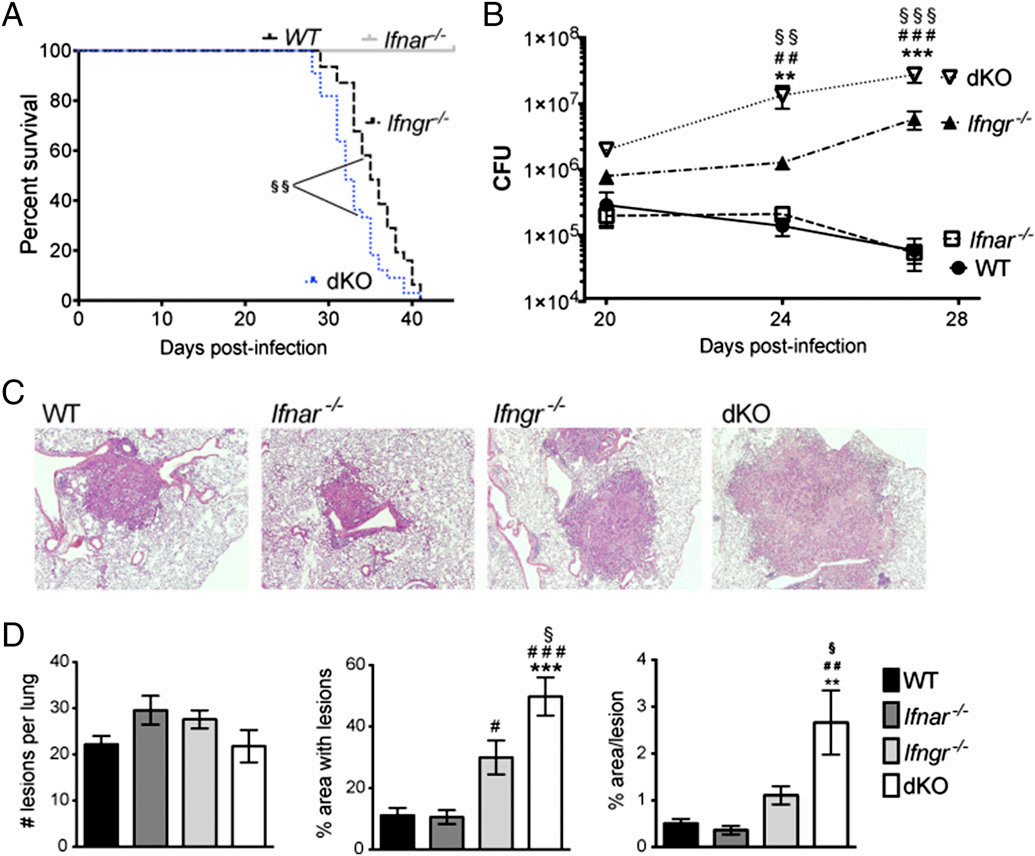
Type I IFN contributes to host protection during infection with virulent *M. tuberculosis* strain in the absence of IFN-γR. WT, *Ifnar^−/−^*, *Ifngr^−/−^*, and *Ifngr*^−/−^ × *Ifnar*^−/−^ (dKO) mice were infected with *M. tuberculosis* strain BTB 02-171. (**A**) Percentage of survival for three independent experiments with 10 mice per group. **^§§^***p* < 0.01, dKO versus *Ifngr^−/−^* mice, log-rank test. (**B**) At the indicated days postinfection, lung cell suspensions were prepared, diluted, and plated onto 7H11 agar to determine the number of mycobacterial CFU in the lungs. (**C** and **D**) H&E-stained tissue of infected lungs at day 27 postinfection was analyzed blindly. (C) Representative images from one of five animals per group (original magnification ×4). (D) Morphometric analysis of the number and size of inflammatory lesions. Each bar represents mean ± SEM for five mice per group. Data are representative of two independent experiments. Significance was determined using two-way ANOVA (B) or one-way ANOVA (D), corrected for multiple comparisons with a Bonferroni test. Significance is shown relative to WT (*), *Ifnar^−/−^* (^#^), or *Ifngr^−/−^* (^§^). *^,^^#^^,^^§^*p* < 0.05, **^,^^##,^^§§^*p* < 0.01, ***^,^^###^^,^^§§§^*p* < 0.001.

**FIGURE 4.**
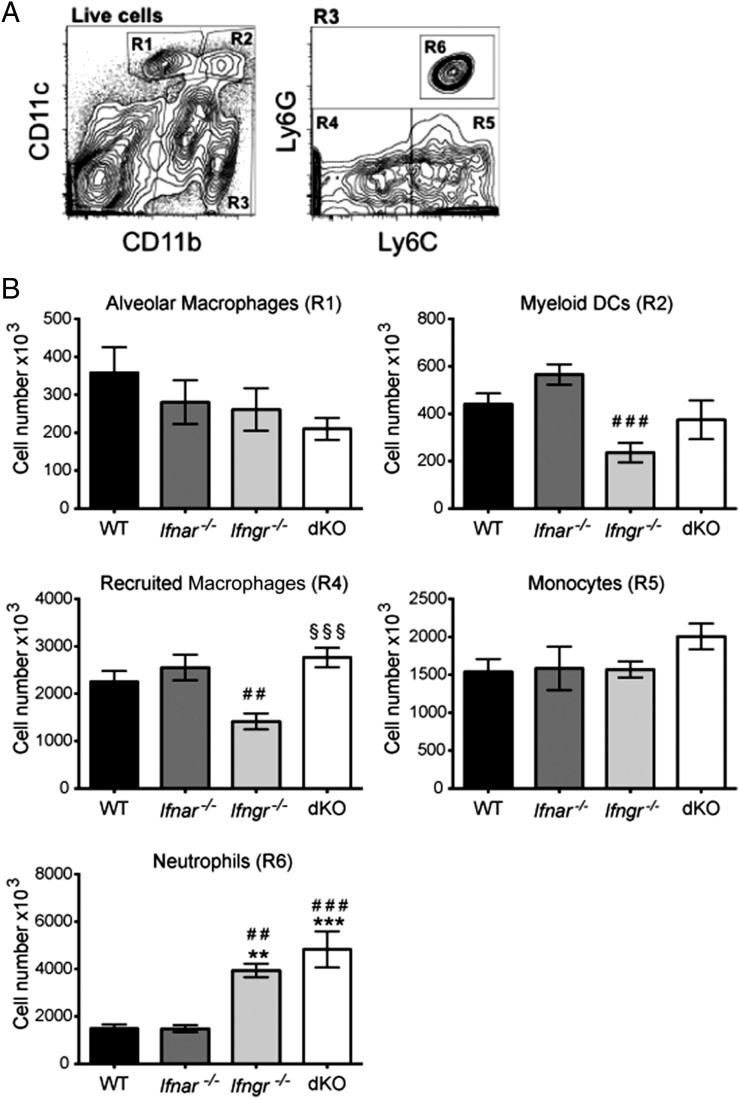
Type I and II IFNs regulate lung myeloid cell recruitment during *M. tuberculosis* infection. WT, *Ifnar^−/−^*, *Ifngr^−/−^*, and *Ifngr*^−/−^ × *Ifnar*^−/−^ (dKO) mice were infected with the *M. tuberculosis* strain BTB 02-17. Myeloid cell populations in infected lungs were characterized by flow cytometry 24 d postinfection. (**A**) Lung cells were gated on single live cells using forward and side scatter parameters. Gating strategy for quantification of myeloid cell populations is shown. R1 (CD11b^lo^ CD11c^+^): alveolar macrophages; R2 (CD11b^hi^ CD11c^+^): myeloid dendritic cells (DCs); gating on R3 (CD11b^hi^ CD11c^neg^), R4 (CD11b^hi^ CD11c^neg^ Ly6C^lo/neg^): recruited macrophages; R5 (CD11b^hi^ CD11c^neg^ Ly6C^int/hi^ Ly6G^neg^): monocytes; and R6 (CD11b^hi^ CD11c^neg^ Ly6C^int^ Ly6G^hi^): neutrophils. (**B**) Cell number of myeloid cell populations. Each bar represents mean ± SEM for five mice per group. Data are a pool of two independent experiments. Significance is shown relative to WT (*), *Ifnar^−/−^* (^#^), or *Ifngr^−/−^* (^§^). **^,^^##^*p* < 0.01, ***^,^^###^*p* < 0.001, one-way ANOVA corrected for multiple comparisons with a Bonferroni test.

The recent discovery of a protective role for type I IFN in *M. tuberculosis* infection in the absence of IFN-γ signaling (39), together with our observation that induction of *Nos2* by type I IFN is masked by the dominant effect of IFN-γ, led us to investigate whether type I IFN might contribute to host protection by inducing macrophage microbicidal mechanisms during *M. tuberculosis* infection in vivo. To address this, we started by assessing the role of type I and type II IFN signaling during infection with *M. tuberculosis* strain BTB 02-171. To this end, *Ifngr*^−/−^ and *Ifnar*^−/−^ mice were intercrossed to obtain *Ifngr*^−/−^ × *Ifnar*^−/−^ (dKO) mice. WT, *Ifnar^−/−^*, *Ifngr^−/−^*, and dKO mice were aerosol infected with a low-dose of virulent *M. tuberculosis* strain BTB 02-171. Similar to the previous study with *M. tuberculosis* strain H37Rv (39), dKO mice succumbed to BTB 02-171 infection significantly earlier than did *Ifngr^−/−^* mice (median survival, 32 versus 35 d, respectively, *p* < 0.0025; Fig. 3A), whereas none of the WT or *Ifnar^−/−^* mice succumbed to disease, even at late stages of infection (surviving >200 d; data not shown). In contrast to what was reported with H37Rv infection, where bacterial loads were no different in dKO mice compared with *Ifngr^−/−^* mice (39), bacterial loads were significantly higher in the lungs of dKO mice compared with *Ifngr^−/−^* mice at days 24 and 27 post–BTB 02-171 infection (Fig. 3B). This suggests that type I IFN also plays a role in controlling bacterial growth in the absence of IFN-γR during in vivo infection with the TLR4-activating *M. tuberculosis* strain BTB 02-171. WT and *Ifnar^−/−^* mice had lower lung bacterial loads than did *Ifngr^−/−^* and dKO mice, and no difference in bacterial loads between WT and *Ifnar^−/−^* mice was observed during the time of the experiment (Fig. 3B) or at later time points (data not shown). Tissue sections of infected lungs were examined to assess pulmonary histology. Increased bacterial loads observed in the absence of both type I and type II IFN receptors were accompanied by enhanced lung pathology at day 27 post–BTB 02-171 infection compared with *Ifngr^−/−^* mice (Fig. 3C, 3D). The extent of the inflammatory infiltrates was significantly greater in dKO mice than in single-KO and WT mice, showing extensive areas of granulomatous inflammation (Fig. 3C, 3D). Enhanced lung pathology correlated with increased numbers of neutrophils in the lungs of dKO and *Ifngr^−/−^* mice, whereas the number of other myeloid cell populations was similar or reduced in the absence of IFN-γR but not in dKO compared with *Ifnar^−/−^* or WT mice (Fig. 4). This is in keeping with the previous report by Desvignes et al. (39) using *M. tuberculosis* strain H37Rv for infection.

### Type I IFN suppresses expression of genes associated with alternatively activated macrophages in *M. tuberculosis*–infected lungs in the absence of IFN-γ signaling

**FIGURE 5.**
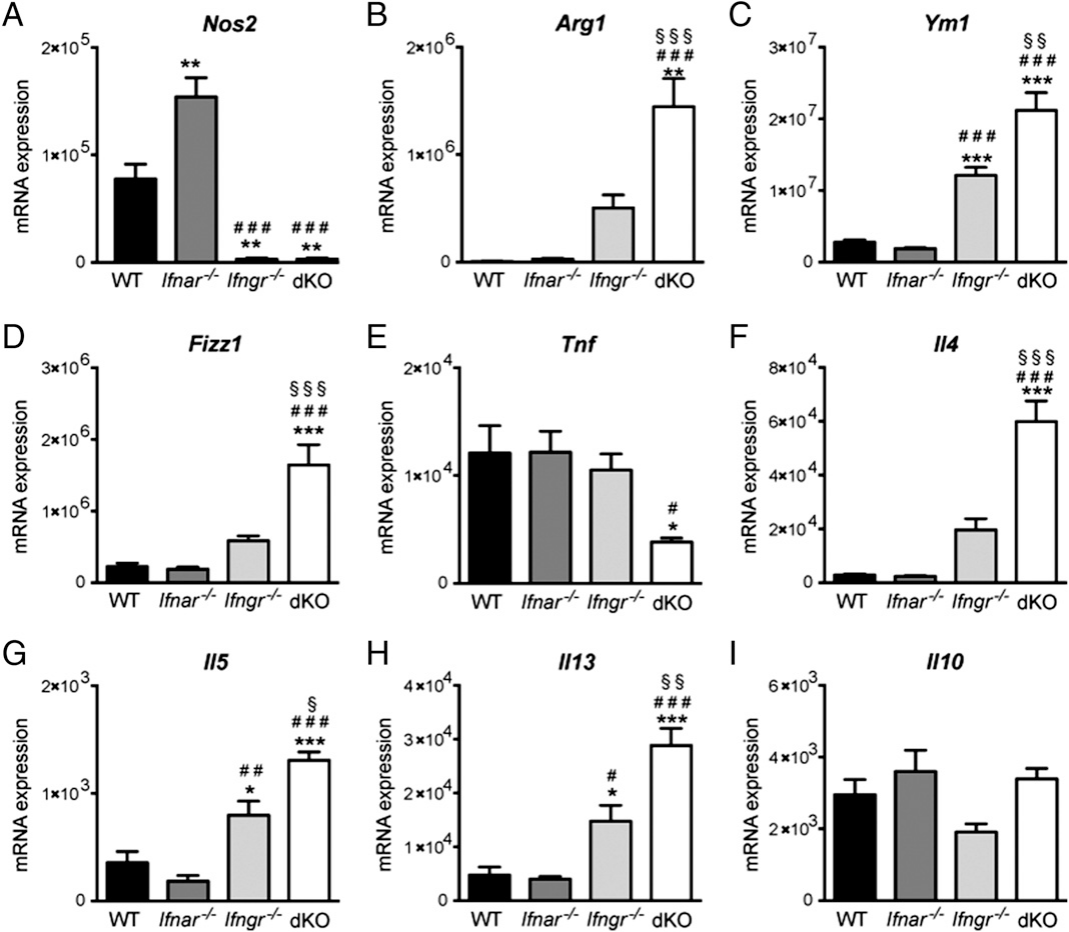
Concomitant loss of type I and type II IFN signaling increases the expression of genes associated with alternatively activated macrophages in *M. tuberculosis*–infected lungs. WT, *Ifnar^−/−^*, *Ifngr^−/−^*, and *Ifngr*^−/−^ × *Ifnar*^−/−^ (dKO) mice were infected with the *M. tuberculosis* strain BTB 02-171. At day 20 postinfection, RNA was extracted from infected lungs and *Nos2* (**A**), *Arg1* (**B**), *Ym1* (**C**), *Fizz1* (**D**), *Tnf* (**E**), *Il4* (**F**), *Il5* (**G**), *Il13* (**H**), and *Il10* (**I**) mRNA expression was analyzed by quantitative real-time PCR and normalized to the expression of *Hprt1*, with the exception of *Tnf*, which was normalized to *Ubiquitin* expression. Each bar represents mean ± SEM for five mice per group. Data are representative of two independent experiments. Significance is shown relative to WT (*), *Ifnar^−/−^* (^#^), or *Ifngr^−/−^* (^§^). *^,^^#^^,^^§^*p* < 0.05, **^,^^##^^,^^§§^*p* < 0.01, ***^,^^###^^,^^§§§^*p* < 0.001, one-way ANOVA corrected for multiple comparisons with a Bonferroni test.

We next examined the contribution of each IFN pathway to the induction of *Nos2* transcription upon in vivo infection with *M. tuberculosis* strain BTB 02-171. As expected, *Nos2* expression was significantly lower in infected lungs from *Ifngr*^−/−^ mice compared with WT mice at day 20 postinfection (Fig. 5A). However, IFNAR signaling did not appear to contribute to *Nos2* induction, because similar or increased levels of *Nos2* were observed between dKO and *Ifngr^−/−^* mice or between *Ifnar^−/−^* and WT mice, respectively (Fig. 5A). Similarly, the number of NOS2-expressing cells in infected lungs was not affected by the loss of IFNAR signaling, but it was greatly reduced in the absence of IFN-γR signaling (Supplemental Fig. 3A). These findings indicate that the protective role of type I IFN during *M. tuberculosis* infection, in the absence of IFN-γ responses, did not correlate with *Nos2* expression in whole infected lungs.

It was shown that *M. tuberculosis* infection induces *Arg1* expression in macrophages, which suppresses NOS2 activity and *M. tuberculosis* killing by these cells (48). Indeed, mice lacking ARG1 exhibit reduced bacterial burden compared with ARG1-competent control mice (48, 49). Because NOS2 and ARG1 are hallmarks of two extremes of macrophage polarization (classically and alternatively activated macrophages, respectively) (50), we hypothesized that the decrease in *Nos2* expression in *Ifngr^−/−^* and dKO mice may be accompanied by an increased expression of *Arg1*. Therefore, we measured *Arg1* gene expression and ARG1 activity in the lungs of WT, *Ifnar^−/−^*, *Ifngr^−/−^*, and dKO mice infected with *M. tuberculosis* strain BTB 02-171. Low levels of *Arg1* expression were detected in the lungs of WT and *Ifnar^−/−^* mice at day 20 post–BTB 02-171 infection (Fig. 5B). Loss of IFN-γR led to an increased expression of *Arg1* in infected lungs (Fig. 5B). Strikingly, loss of both type I and type II IFNRs resulted in significantly higher *Arg1* expression than that observed in the absence of IFN-γR alone (Fig. 5B). Similarly, ARG1 activity was significantly enhanced in the lungs of infected dKO mice compared with *Ifngr^−/−^* mice (Supplemental Fig. 3B), indicating that type I IFN suppresses *Arg1* gene expression and ARG1 activity in the absence of IFN-γR. In addition, increased pulmonary expression of other markers associated with alternatively activated macrophages, *Ym1* and *Fizz1*, was detected in dKO mice compared with *Ifngr^−/−^*, *Ifnar^−/−^*, and WT mice (Fig. 5C, 5D).

To further understand the molecular basis for the switch between classically activated and alternatively activated macrophages observed during *M. tuberculosis* infection in the absence of IFNAR and IFN-γR, we measured the expression of several cytokines previously associated with the control of macrophage polarization (50, 51). Coincident with the increased expression of genes associated with alternatively activated macrophages (Fig. 5B–D), we found decreased expression of *Tnf*, a cytokine associated with classically activated macrophages (50, 51), in the lungs of infected dKO mice compared with *Ifngr^−/−^*, *Ifnar^−/−^*, and WT mice (Fig. 5E). Furthermore, the presence of markers associated with alternatively activated macrophages also coincided with an increase in mRNA expression of Th2 cytokines, such as IL-4, IL-5, and IL-13, but not IL-10, in infected lungs in the absence of IFN-γR that was enhanced significantly by the loss of IFNAR in the absence of IFN-γR (Fig. 5F–I).

Our data demonstrate that, in the absence of IFN-γ signaling, additional loss of type I IFN signaling results in very high levels of expression of markers associated with alternatively activated macrophages in infected lungs, which likely suppress bacterial killing.

### Type I IFN suppresses Arg1 expression in *M. tuberculosis*–infected macrophages

**FIGURE 6.**
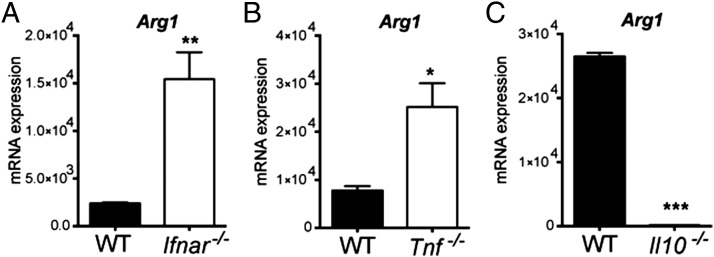
Type I IFN and TNF suppress *Arg1* transcription in macrophages infected with *M. tuberculosis*. WT and *Ifnar^−/−^* (**A**), WT and *Tnf^−/−^* (**B**), or WT and *Il10^−/−^* (**C**) macrophages were infected with BTB 02-171 (MOI = 2), and *Arg1* mRNA levels were determined by quantitative real-time PCR at 6 h postinfection and normalized to the expression of *Hprt1*. Graphs show mean ± SEM of triplicate samples. Data are representative of at least two independent experiments. *p < 0.05, **p < 0.01, ***p < 0.001, unpaired *t* test.

**FIGURE 7.**
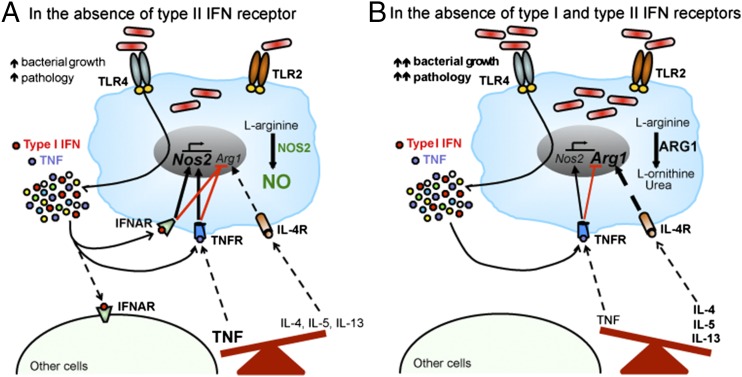
Schematic summary of the regulation of macrophage activation by type I IFN during *M. tuberculosis* infection. (**A** and **B**) Activation of TLR4 in macrophages by specific *M. tuberculosis* strains leads to the production of type I IFN, in addition to other cytokines (e.g., TNF) (15). Type I IFN induces *Nos2* and inhibits *Arg1* gene expression and protein activity in infected macrophages, thus regulating macrophage activation toward a more protective phenotype. Induction of *Nos2* and suppression of *Arg1* transcription in infected macrophages are further potentiated by macrophage-derived TNF. In vivo, in the absence of IFN-γ signaling, type I IFN suppresses the expression of markers associated with alternatively activated macrophages following *M. tuberculosis* infection, likely by direct regulation of macrophage activation, as well as by modulating TNF and Th2-associated cytokine expression, contributing to host protection.

To evaluate whether macrophage-derived type I IFN could have a direct effect on suppressing *Arg1* induction in response to *M. tuberculosis* infection, we infected WT and *Ifnar^−/−^* macrophages with *M. tuberculosis* strain BTB 02-171 and examined the subsequent effects on *Arg1* mRNA expression. This revealed that macrophage-derived type I IFN suppressed *Arg1* induction in infected macrophages, because *Arg1* levels were significantly higher in *Ifnar^−/−^* macrophages than in WT macrophages at 6 h post–BTB 02-171 infection (Fig. 6A). Macrophage-derived TNF also suppressed *Arg1* induction in infected macrophages, because *Arg1* levels were significantly higher in *Tnf^−/−^* macrophages compared with WT macrophages (Fig. 6B). Although loss of type I IFN signaling decreased *Tnf* expression during in vivo infection in the absence of IFN-γR (Fig. 5E), TNF production in response to *M. tuberculosis* infection in vitro was not significantly different between WT and *Ifnar^−/−^* macrophages (Supplemental Fig. 4A). Therefore, both type I IFN and TNF suppressed *Arg1* expression in infected macrophages, although the inhibition of *Arg1* expression by type I IFN was not accompanied by increased levels of TNF production in vitro. In contrast, macrophage-derived IL-10 induced *Arg1* expression in infected macrophages, because *Arg1* levels were significantly reduced in *Il10^−/−^* macrophages compared with WT macrophages (Fig. 6C). Because type I IFN and TNF induced *Nos2* gene expression and NOS2 activity in *M. tuberculosis*–infected macrophages (Fig. 2E, Supplemental Fig. 2H, 2K, 2L), we assessed whether suppression of *Arg1* expression by type I IFN and TNF was dependent on NOS2. Similar levels of *Arg1* expression were detected in *Nos2^−/−^* and WT macrophages infected with the BTB 02-171 strain (Supplemental Fig. 4B), indicating that suppression of *Arg1* transcription by type I IFN and TNF appeared to be independent of *Nos2* induction or NOS2 activity. Taken together, our results show that type I IFN might inhibit the differentiation of alternatively activated macrophages by direct downregulation of *Arg1* transcription in *M. tuberculosis*–infected macrophages, although other factors further potentiate this mechanism in vivo (Fig. 7).

## Discussion

Type I IFN plays a role in *M. tuberculosis* persistence and TB pathogenesis (16–30, 32, 33). However, in particular cases, such as in multidrug resistant TB patients treated with antimycobacterial drugs (52, 53) or in patients with mutations in their IFN-γR suffering from mycobacterial infections (40, 54), type I IFN could provide some level of protection. Thus, unraveling the molecular mechanisms underlying this protective role may offer new targets for host-direct therapies in these individuals. In the mouse model of infection, a protective role was attributed to type I IFN, in the absence of IFN-γ–dependent immunity, by influencing the recruitment and/or survival of potential target cells in infected lungs (39). In this article, we describe a previously unappreciated role for type I IFN in regulating macrophage activation during *M. tuberculosis* infection, revealing a novel mechanism by which type I IFN may confer protection against *M. tuberculosis* infection in the absence of IFN-γ signaling (Fig. 7). Our findings demonstrate that type I IFN induces *Nos2* and inhibits *Arg1* expression following infection of macrophages with *M. tuberculosis*, resulting in high NO production by infected macrophages. The suppression of *Arg1* expression by type I IFN, and indeed of other genes associated with alternatively activated macrophages, was also observed during in vivo infection in the absence of IFN-γ signaling. These alterations correlated with control of bacterial growth and pathology. Taken together, our data show that, although masked by the dominant effect of IFN-γ, type I IFN has a regulatory function in macrophage activation during *M. tuberculosis* infection; it contributes to host protection by suppressing the switching of macrophages from a more protective, classically activated phenotype to a more permissive, alternatively activated phenotype.

Mutations in both IFN-γR chains, *IFNGR1* and *IFNGR2*, were identified in humans and have been associated with increased susceptibility to mycobacterial infections (9). It was reported that adjunctive treatment with IFN-α confers clinical benefits in patients with mutations in the IFN-γR who are suffering from mycobacterial diseases (40, 54). Our results provide important information that may help to explain the mechanism underlying the beneficial effect of IFN-α treatment and provide novel targets for host-directed therapy to improve future treatment of patients with IFN-γR mutations or with compromised IFN-γ responses.

NO synthesis by NOS2 is critical for effective immunity and host protection against virulent strains of *M. tuberculosis* (12–14, 41, 55). Although *M. tuberculosis* strain BTB 02-171 induces strong *Nos2* expression early after in vivo infection, this strain still causes severe disease, as shown by increased bacterial loads and enhanced lung pathology (15). In some bacterial infections, an exacerbated expression of NOS2 was associated with a more severe disease outcome, likely due to NO-mediated cytotoxicity and tissue damage and suppression of the immune response to the pathogen (43–45). Although we cannot completely exclude the hypothesis that elevated *Nos2* expression can contribute to disease severity during infection with *M. tuberculosis* strain BTB 02-171, mice deficient for NOS2 were extremely susceptible to infection with this strain. Additionally, compared with the less virulent laboratory strain H37Rv, *Nos2^−/−^* mice showed earlier susceptibility to BTB 02-171 infection that correlates with earlier induction of elevated levels of *Nos2* expression following infection with this strain. Temporal differences in *Nos2* induction following infection with different strains of *M. tuberculosis* may explain previous reports showing a protective role for NOS2 during the late, but not the early, phase of infection (41).

Surprisingly, we found that the expression of *Nos2* in macrophages infected with *M. tuberculosis* strain BTB 02-171 was independent of the presence of IFN-γ, a key cytokine for activation of macrophages during *M. tuberculosis* infection to produce large amounts of NO (11, 13, 47, 56). We then investigated the molecular mechanisms underlying this IFN-γ–independent *Nos2* induction and NO production and found a requirement for TLR4/TRIF signaling. Type I IFN was reported to contribute to the induction of NO production following stimulation of macrophages with the TLR4 ligand LPS (57). We found that type I IFN signaling was required for transcriptional induction of *Nos2* and consequent NO production in macrophages infected with BTB 02-171. Furthermore, addition of IFN-β significantly enhanced NO production following infection with the H37Rv strain, which, on its own, induced little production of NO by infected macrophages.

Nevertheless, our results show that IFN-γ is a stronger inducer of NO production by *M. tuberculosis*–infected macrophages than type I IFN, likely due to prolonged transcriptional induction of *Nos2*. Furthermore, IFN-γ–induced NOS2, but not type I IFN–induced NOS2, appears to be required for efficient bacterial control by macrophages. We reported previously that type I IFN impairs IFN-γ’s effects on cytokine production by macrophages infected with *M. tuberculosis* (33). Type I IFN completely abrogated the ability of IFN-γ to enhance IL-12 and TNF production and to inhibit IL-10 production by macrophages in response to H37Rv infection, although it did not impair IFN-γ inhibition of IL-1β production (33). We now show that type I IFN can also downregulate IFN-γ–dependent induction of NO production by infected macrophages. Because IFN-γ–dependent inhibition of IL-1β production during *M. tuberculosis* infection seems to be mediated by *Nos2* (33, 58), our recent finding may explain why the ability of IFN-γ to suppress IL-1β production by infected macrophages was indeed increased by the presence of type I IFN (33). Although IL-10 is an important mediator of the suppressive effect of type I IFN on IL-12 and TNF macrophage production induced by IFN-γ, IL-10 did not play a role in suppressing the IFN-γ–dependent induction of NO production by *M. tuberculosis*–infected macrophages (data not shown).

We report in this article that, in vivo, *Nos2* expression required IFN-γ signaling because *Nos2* mRNA levels were significantly lower in infected lungs from mice deficient for IFN-γR compared with WT mice, in agreement with previous reports (11, 13, 32). Type I IFN inhibited IFN-γ*–*induced *Nos2* transcription slightly, in line with our in vitro data and with another study reporting an IFNAR-mediated inhibition of IFN-γ–induced NOS2 expression in lung myeloid cells (32). However, type I IFN–dependent *Nos2* induction was not detected in infected lungs at the time points analyzed postinfection. One explanation is that type I IFN–induced *Nos2* transcription is transient and not sustained over time, in contrast to IFN-γ–dependent *Nos2* induction. This hypothesis is supported by our in vitro data showing that, although both type I and type II IFNs induced similar levels of *Nos2* early following macrophage infection, increased levels of *Nos2* were only sustained over time in the presence of IFN-γ. *Nos2* induction was reported to be critical to control neutrophil-mediated pulmonary pathology following *M. tuberculosis* infection (58). Therefore, induction of *Nos2* by type I IFN (even if transient or in low levels) could explain the increased numbers of neutrophils and enhanced lung pathology observed in the absence of both IFN-γR and IFNAR. In addition to enhanced pathology, we observed increased lung bacterial loads in the absence of both IFN-γR and IFNAR, compared with the absence of IFN-γR alone, which contrasts with a previous study of *M. tuberculosis* strain H37Rv (39). The strain of *M. tuberculosis* used in this study, BTB 02-171, is more virulent and induces higher levels of type I IFN in the lungs of infected mice than does the H37Rv strain (15), which may account for some of the differences observed between the previous study and ours (39).

The increased susceptibility to *M. tuberculosis* infection that we observed in the absence of both IFN-γR and IFNAR signaling correlated with increased expression levels of genes associated with alternatively activated macrophages, such as *Arg1*, *Ym1*, and *Fizz1*, in the lungs. A positive correlation between ARG1 and human TB was suggested recently based on the increased expression of *ARG1* in monocytes isolated from peripheral blood of patients with active TB compared with those with latent TB (49). ARG1 was also reported to be expressed in granuloma-associated macrophages of lung tissues from patients with TB (59). Likewise, *M. tuberculosis* infection can induce *Arg1* expression in murine macrophages (48, 60), and specific elimination of *Arg1* in macrophages decreased lung bacterial loads during in vivo infection (48, 49). How macrophage expression of *Arg1* during *M. tuberculosis* infection increases susceptibility to *M. tuberculosis* infection remains unclear. It was suggested that ARG1 impairs bacterial growth restriction by infected macrophages by suppressing NOS2 activity and preventing NO production (48), which could be part of the mechanism observed in our study of *M. tuberculosis* infection in the absence of IFN-γR and IFNAR signaling. As another mechanism, we consider the possibility that ARG1 activity may supply substrates for *M. tuberculosis* growth and survival, as was suggested for *Leishmania* species (61).

Alternative macrophage activation is typically induced by IL-4Rα activation (50, 51). Differentiation of alternatively activated macrophages with IL-4 in vitro was reported to inhibit macrophage antimicrobial responses to *M. tuberculosis* (62). Moreover, Th2 cell responses were associated with TB pathogenesis by mediating the alternative activation of macrophages during *M. tuberculosis* infection (49, 63, 64). We detected increased levels of Th2-associated cytokines in infected lungs in the absence of IFN-γR, which were further enhanced by the concomitant absence of IFNAR, suggesting that increased IL-4Rα signaling may drive alternative activation of lung macrophages in *Ifngr^−/−^* and dKO mice. In addition, decreased levels of TNF expression were detected in the absence of both IFN-γR and IFNAR. TNF was recently shown to hamper alternative activation of macrophages in murine models of cancer by suppressing Th2-associated cytokine expression (65). Although the effect of TNF on the expression of Th2-associated cytokines remains to be clarified in our in vivo model, our data show that TNF directly inhibits *Arg1* expression in *M. tuberculosis*–infected macrophages. In contrast, IL-10 is required for maximal induction of *Arg1* expression in *M. tuberculosis*–infected macrophages in vitro. Overexpression of IL-10 by macrophages and monocytes (under control of the CD68 promoter) was shown to induce the expression of *Arg1* and other markers associated with alternative macrophage activation during *M. tuberculosis* infection, increasing host susceptibility to TB (66). However, similar levels of IL-10 expression were detected in infected lungs in the presence or absence of IFN-γR and IFNAR, suggesting that IL-10 does not play a major role in the regulation of *Arg1* expression in our in vivo model. Type I IFN inhibits *Arg1* expression by infected macrophages in our in vitro model, in which Th2-associated cytokines are absent, and TNF production was not affected by the absence of IFNAR. These findings point to a direct suppressor function of type I IFN on the transcriptional induction of *Arg1* in macrophages following *M. tuberculosis* infection. Therefore, during in vivo infection, *Arg1* expression may be directly inhibited by type I IFN signaling and indirectly inhibited by type I IFN–dependent regulation of TNF and Th2-associated cytokine expression.

In summary, our findings demonstrate that inhibition of alternative macrophage activation by type I IFN correlates with control of bacterial growth and pathology during infection with a virulent strain of *M. tuberculosis* in the absence of IFN-γR. Type I IFN inhibits transcriptional induction of *Arg1* in infected macrophages. Moreover, in the absence of IFN-γR, IFNAR signaling inhibits the expression of Th2-associated cytokines and enhances TNF expression in infected lungs, which likely further contributes to the suppression of alternative macrophage activation in vivo. In addition to furthering our understanding of the modulation of macrophage activation during *M. tuberculosis* infection, these data provide evidence for a novel mechanism by which type I IFN, in the absence of IFN-γR, may confer protection against *M. tuberculosis* infection; this offers new avenues to develop host-direct therapies for patients with compromised IFN-γ responses.

## Supplementary Material

Data Supplement
