# Supplementary material for: Type I IFN Inhibits Alternative Macrophage Activation during Mycobacterium tuberculosis Infection and Leads to Enhanced Protection in the Absence of IFN-γ Signaling
Source: J Immunol. 2016 Nov 14;197(12):4714–26. doi: 10.4049/jimmunol.1600584 (PMC5133670; doi:10.4049/jimmunol.1600584)
Supplement: Data Supplement [file JI_1600584.zip › JI_1600584_Supplemental_Figures_1.pdf]

Supplemental Information

Type I IFN inhibits alternative macrophage activation during *Mycobacterium tuberculosis* infection and leads to enhanced protection in the absence of IFN-γ signaling

Lúcia Moreira-Teixeira, Jeremy Sousa, Finlay W McNab, Egídio Torrado, Filipa Cardoso, Henrique Machado, Flávia Castro, Vânia Cardoso, Joana Gaifem, Xuemei Wu, Rui Appelberg, António Gil Castro, Anne O'Garra and Margarida Saraiva

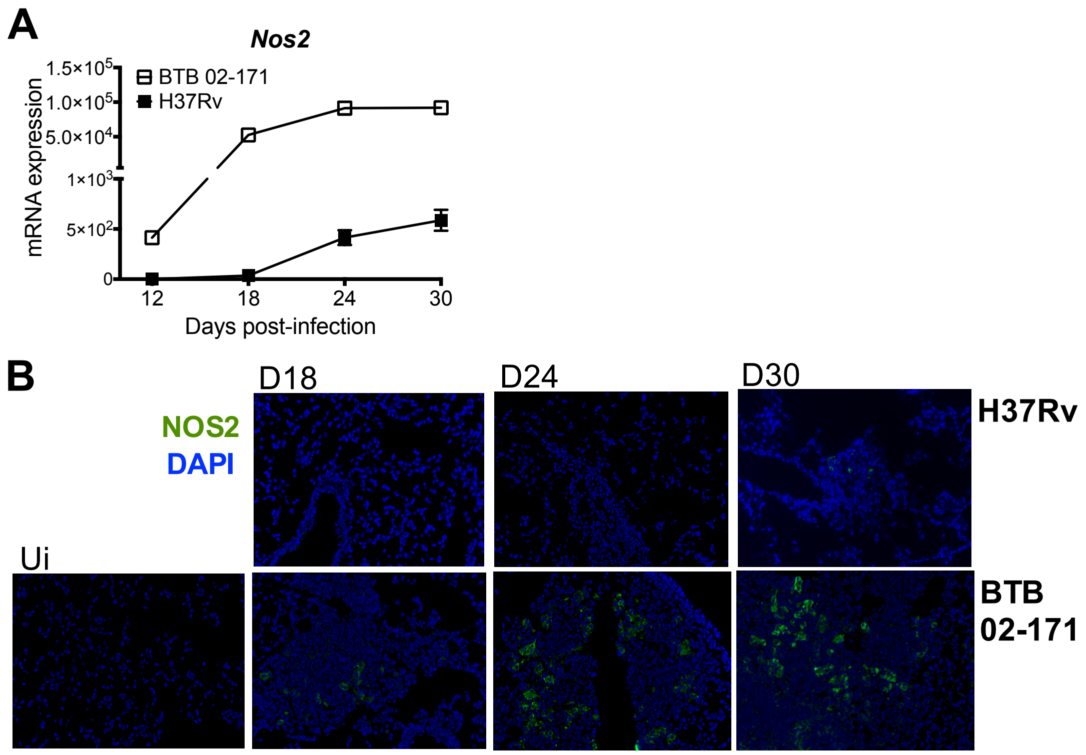

Supplemental Figure 1. Virulent *M. tuberculosis* strain BTB 02-171 induces high levels of NOS2 in infected lungs early after infection. WT mice were infected with *M. tuberculosis* strains H37Rv (closed squares) or BTB 02-171 (open squares). (**A**) At indicated days post-infection, RNA was extracted from infected lungs and *Nos2* expression analyzed by qRT-PCR and normalized to the expression of *Hprt1*. Data points show the mean±SEM for 5 mice per group. (**B**) Expression of NOS2 in the lung tissue of uninfected (Ui) or infected mice was determined by immunofluorescence (green signal indicates NOS2 stain and blue signal indicates cell nuclei). Represented are 4x magnifications of one animal out of 3 per group. Data are from one independent experiment for each *M. tuberculosis* strain.

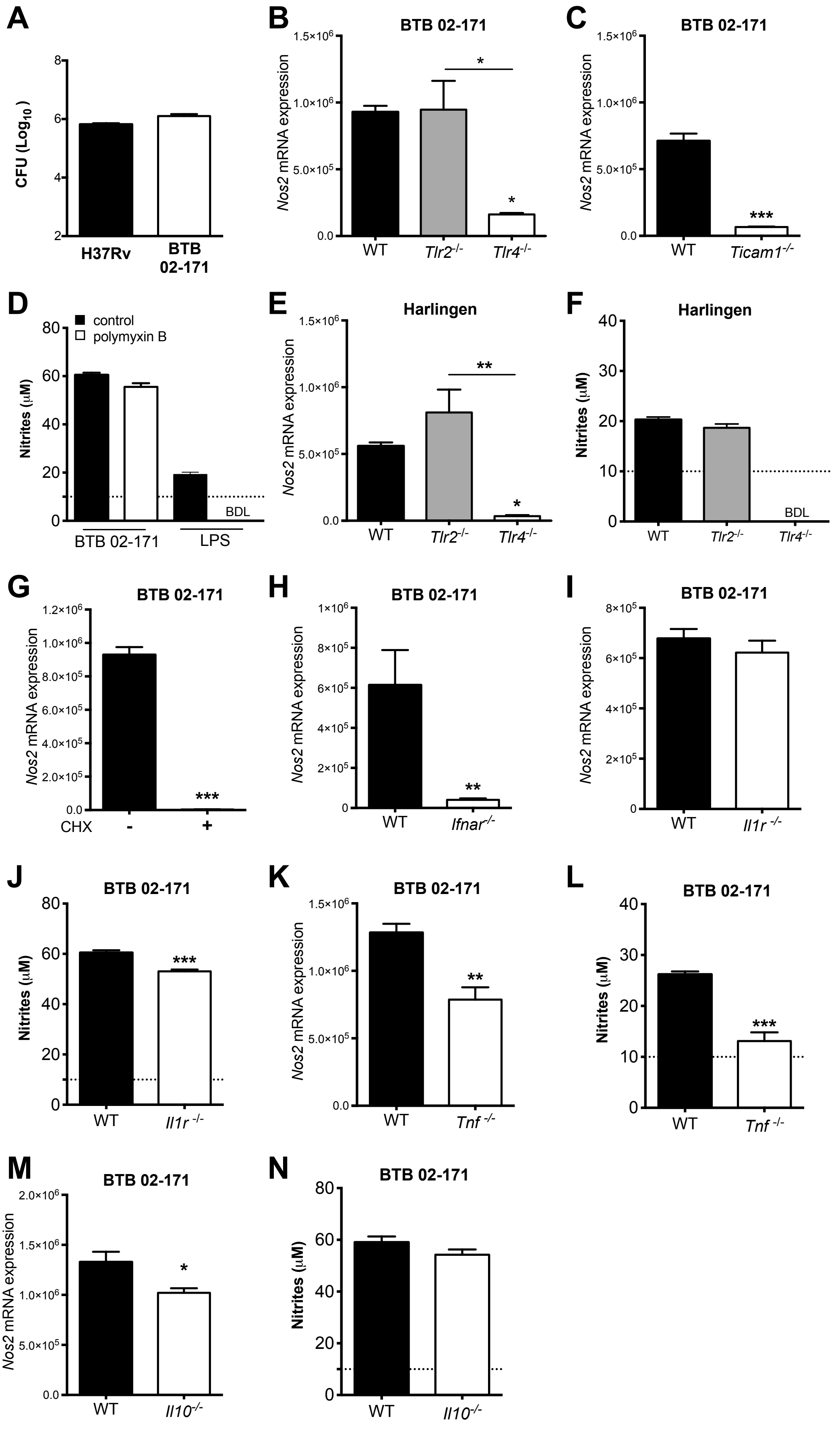

A
CFU (Log10)
H37Rv
BTB 02-171
B
BTB 02-171
Nos2 mRNA expression
WT
Tlr2-/-
Tlr4-/-
C
BTB 02-171
Nos2 mRNA expression
WT
Ticam1-/-
D
control
polymyxin B
Nitrites (μM)
BDL
BTB 02-171
LPS
E
Harlingen
Nos2 mRNA expression
WT
Tlr2-/-
Tlr4-/-
F
Harlingen
Nitrites (μM)
BDL
WT
Tlr2-/-
Tlr4-/-
G
BTB 02-171
Nos2 mRNA expression
CHX
H
BTB 02-171
Nos2 mRNA expression
WT
Ifnar-/-
I
BTB 02-171
Nos2 mRNA expression
WT
Il1r -/-
J
BTB 02-171
Nitrites (μM)
WT
Il1r -/-
K
BTB 02-171
Nos2 mRNA expression
WT
Tnf -/-
L
BTB 02-171
Nitrites (μM)
WT
Tnf -/-
M
BTB 02-171
Nos2 mRNA expression
WT
Il10-/-
N
BTB 02-171
Nitrites (μM)
WT
Il10-/-

Supplemental Figure 2. Macrophage-derived type I IFN induces *Nos2* expression in macrophages infected with TLR4-activating *M. tuberculosis* strains. (**A**) WT macrophages were infected with *M. tuberculosis* strains H37Rv or BTB 02-171 for 4h (MOI=2). Cells were then extensively washed and lysed and the bacterial burden determined by CFU counting in 7H11 agar plates. (**B** and **C**) WT, *Tlr2*$^{-/-}$ and *Tlr4*$^{-/-}$ (**B**) or WT and *Ticam*$^{-/-}$ (**C**) macrophages were infected with BTB 02-171 and *Nos2* mRNA levels were determined by qRT-PCR at 6h post-infection and normalized to the expression of *Hprt1*. (**D**) WT macrophages were infected with BTB 02-171 or stimulated with 0.5μg/ml LPS in the presence or absence of 5μg/ml polymyxin B. NO production was determined by Griess reagent assay of nitrites in culture supernatants at 24h post-infection. (**E** and **F**) WT, *Tlr2*$^{-/-}$ and *Tlr4*$^{-/-}$ macrophages were infected with *M. tuberculosis* strain Harlingen. (**E**) *Nos2* mRNA levels were determined by qRT-PCR at 6h. (**F**) NO levels in culture supernatants were determined by Griess reagent assay at 24h. (**G**) WT macrophages were infected with BTB 02-171 in the presence or absence of 10μg/ml cycloheximide (CHX) and *Nos2* mRNA levels were determined by qRT-PCR at 6h. (**H**) WT and *Ifnar*$^{-/-}$ macrophages were infected with BTB 02-171 and *Nos2* mRNA levels were determined by qRT-PCR at 6h. (**I-N**) WT and *Il1r*$^{-/-}$ (**I** and **J**) or WT and *Tnf*$^{-/-}$ (**K** and **L**) or WT and *Il10*$^{-/-}$ (**M** and **N**) macrophages were infected with BTB 02-171. (**I**, **K** and **M**) *Nos2* mRNA levels were determined by qRT-PCR at 6h. (**J, L** and **N**) NO levels in culture supernatants were determined by Griess reagent assay at 24h. Graphs show mean±SEM of triplicate samples, except for (**A**) which shows data from 6 wells. Data are from one (**A-D**, **F**, **G**, **I**), two (**E**, **J-N**) or three (**H**) independent experiments. Significance was determined using one-way ANOVA with Bonferroni correction test (**B**, **E**) or unpaired *t* test (**C**, **G-N**). Significance relative to control group, unless otherwise indicated in the figure. *$p<0.05$, **$p<0.01$, ***$p<0.001$.

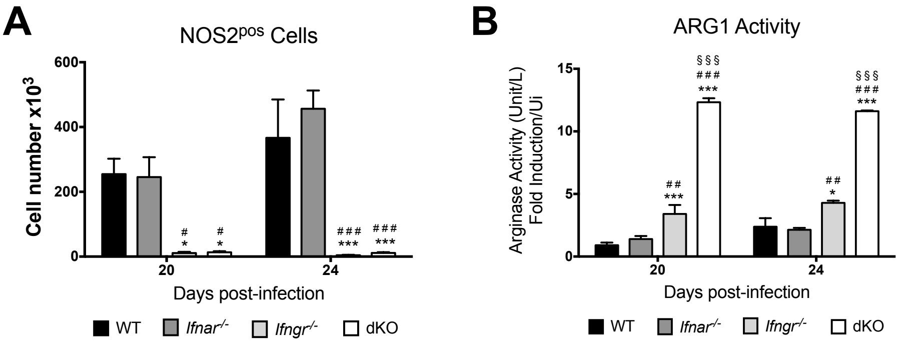

Supplemental Figure 3. Protective role of type I IFN during *M. tuberculosis* infection, in the absence of IFN-γR, correlates with lower ARG1 activity in infected lungs. WT, *Ifnar*$^{-/-}$, *Ifngr*$^{-/-}$ and *Ifngr*$^{-/-}$ x *Ifnar*$^{-/-}$ (dKO) mice were infected with the *M. tuberculosis* strain BTB 02-17. (**A**) At the indicated days post-infection, lung cell suspensions were prepared and total NOS2 expressing cells were determined by flow cytometry (gated on single live cells using forward and side scatter parameters). (**B**) ARG1 activity was measured as urea production by lysed lung cells after addition of L-arginine. Each bar represents mean±SEM for 5 mice per group. Data are from one experiment. Significance was determined using two-way ANOVA corrected for multiple comparisons with a Bonferroni test. Significance is shown relative to WT (*), *Ifnar*$^{-/-}$ (#) or *Ifngr*$^{-/-}$ (§). *p<0.05, **p<0.01, ***p<0.001.

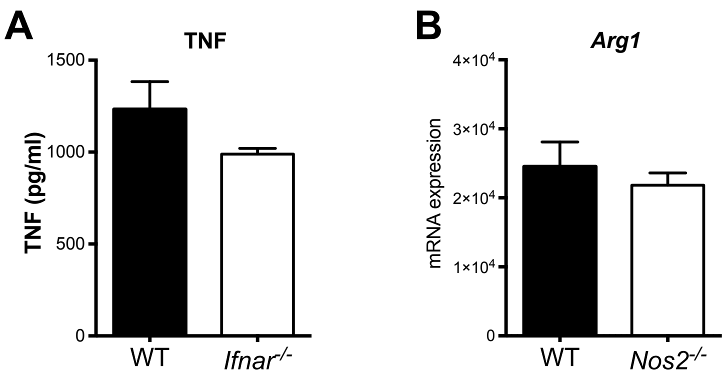

Supplemental Figure 4. Absence of NOS2 has no impact on *Arg1* expression in macrophages infected with virulent *M. tuberculosis* strain BTB 02-171. (**A**) WT and *Ifnar*$^{-/-}$ macrophages were infected with BTB 02-171 (MOI=2) and levels of TNF in culture supernatants were determined by ELISA at 24h post-infection. (**B**) WT and *Nos2*$^{-/-}$ macrophages were infected with BTB 02-171 (MOI=2) and *Arg1* mRNA levels were determined by qRT-PCR at 6h post-infection and normalized to the expression of *Hprt1*. Graphs show mean±SEM of triplicate samples. Data are representative of two (**A**) or one (**B**) experiments.
